# Age at Time of Kidney Transplantation as a Predictor for Mortality, Graft Loss and Self-Rated Health Status: Results From the Swiss Transplant Cohort Study

**DOI:** 10.3389/ti.2021.10076

**Published:** 2022-01-27

**Authors:** Nadine Beerli, Kris Denhaerynck, Isabelle Binet, Suzan Dahdal, Michael Dickenmann, Delaviz Golshayan, Karine Hadaya, Uyen Huynh-Do, Aurelia Schnyder, Sabina M. De Geest, Oliver Mauthner

**Affiliations:** ^1^ Institute of Nursing Science, University of Basel, Basel, Switzerland; ^2^ University Department of Geriatric Medicine Felix Platter, Basel, Switzerland; ^3^ Clinic of Nephrology and Transplantation Medicine, Cantonal Hospital St. Gallen, St. Gallen, Switzerland; ^4^ University Clinic for Nephrology and Hypertension, University Hospital Bern, Bern, Switzerland; ^5^ Department for Transplantation-Immunology and Nephrology, University Hospital Basel, Basel, Switzerland; ^6^ Transplantation Centre and Transplantation Immunopathology Laboratory, University Hospital Lausanne, Lausanne, Switzerland; ^7^ Department of Nephrology, University Hospital Geneva, Geneva, Switzerland; ^8^ Academic Centre for Nursing and Midwifery, Department of Public Health and Primary Care, KU Leuven, Leuven, Belgium

**Keywords:** mortality, renal transplantation, age, graft loss, end stage renal disease, patient reported outcome measures

## Abstract

**Introduction:** The effect of age on health outcomes in kidney transplantation remains inconclusive. This study aimed to analyze the relationship between age at time of kidney transplantation with mortality, graft loss and self-rated health status in adult kidney transplant recipients.

**Methods:** This study used data from the Swiss Transplant Cohort Study and included prospective data of kidney transplant recipients between 2008 and 2017. Time-to-event analysis was performed using Cox’ regression analysis, and -in the case of graft loss- competing risk analysis. A random-intercept regression model was applied to analyse self-rated health status.

**Results:** We included 2,366 kidney transplant recipients. Age at transplantation linearly predicted mortality. It was also predictive for graft loss, though nonlinearly, showing that recipients aged between 35 and 55 years presented with the lowest risk of experiencing graft loss. No relationship of age with self-rated health status was detected.

**Conclusion:** Higher mortality in older recipients complies with data from the general population. The non-linear relationship between age and graft loss and the higher scored self-rated health status at all follow-up time-points compared to the pre-transplant status -regardless of age- highlight that age alone might not be an accurate measure for risk prediction and clinical decision making in kidney transplantation.

## Graphical Abstract

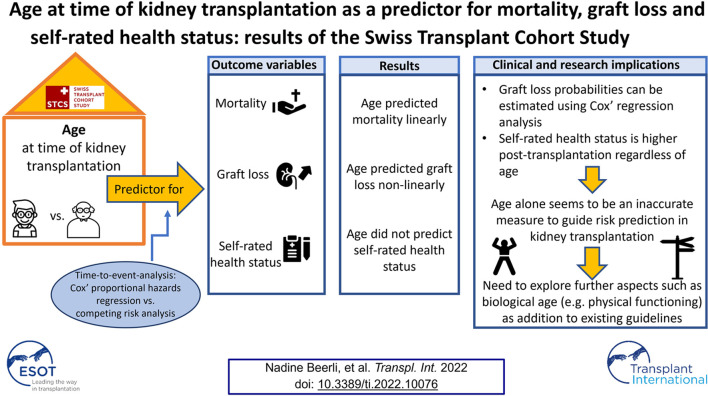

## Introduction

Ageing populations and a higher incidence of chronic conditions with advanced age have resulted in increasing numbers of older patients with end-stage renal disease (ESRD) [1, 2]. This trend is supported by a growing group of older adults considered eligible for and undergoing kidney transplantation (KT) [3–6]. According to records from the Swiss Transplant Cohort Study (STCS), 21% of all KT recipients in Switzerland—where there is no age limit prohibiting access to KT—are ≥65 years of age at time of transplantation [7]. In this context, age always refers to chronological age, i.e., the age counted in years since date of birth. Recently published guidelines recommend considering all patients with chronic kidney disease who are likely to progress to ESRD for KT regardless of their age [8]. KT is considered the preferred treatment option compared to hemodialysis, as it provides better results in terms of survival, cost effectiveness and patient reported quality of life [8–11]. The demand for KT at the same time significantly exceeds the number of available donor organs, thus, studies focusing on predictors for outcomes in older KT recipients present an important research area to support clinical decision and policy making.

Older patients often present with conditions such as disability, functional and cognitive decline and increased numbers of comorbidities such as cardiopulmonal diseases, diabetes or cancer, which can result in adverse health outcomes. Most studies point at an increased post-KT mortality in older KT recipients, an expected finding when comparing outcomes with study results from the general population [12–16]. On the contrary, a number of studies reported mortality rates similar to or lower than in adults of younger age [17–19]. Moreover, patients undergoing KT show a lower mortality risk compared to similar patients remaining on the waitlist and on dialysis [3, 20–22]. Inconsistencies also exist for graft loss, with studies showing higher rates in the older cohort [14, 17, 23] or alternatively a non-significant or protective effect by increasing age [15, 18, 24, 25].

Further, to better understand the effectiveness of healthcare interventions, the inclusion of patient reported outcome measures (PROMs) in addition to more commonly studied biomedical outcomes such as mortality and graft loss, is increasingly being acknowledged in transplant research [26]. In KT PROMs, like quality of life and self-rated health status, have been found to improve pre- to post-transplant in all age groups. Prospective longitudinal data from larger data sets, however, are scarce [9, 26]. Thus, studies in KT that include PROMs to better evaluate health outcomes over time are needed.

In previous studies, two methodological limitations in the field of KT point to the need to improve applied methods in future research. First, age has been frequently used as a categorical variable to facilitate interpretation of study findings, with varying age cut-offs across studies, thus assuming non-linear relationships. However, no one has investigated whether this holds true and where such a relationship would divert from linearity. Second, mortality and graft loss in KT are commonly analyzed using standard survival analysis (e.g., Kaplan-Meier survival curves and Cox’ proportional hazards regression). These methods only take into account one type of outcome per analysis, whereas KT recipients are simultaneously at risk for several adverse events. When graft survival is analyzed, a patient can experience death with a functioning graft, without altering the probability of graft loss, typically resulting in overestimated outcome probabilities [27].

Clinicians and policymakers have to rely on a limited body of evidence to guide organ allocation as well as pre- and post-KT management for older recipients. Thus, prospective multi-center research is essential to provide insights regarding causal relationships between patient’s age and post-KT outcomes, with potential for generalizability [5, 6, 8, 11, 20, 28]. In particular, since age is still associated with lower odds to be waitlisted for and access to KT [12, 24, 29]. The aim of this study was to analyze the relationship between age at time of KT with mortality, graft loss incidence and post-transplant self-rated health status in adult KT recipients while controlling for bio-psychosocial risk factors.

## Patients and Methods

### Design, Setting and Sample

This study used data from the STCS, a nation-wide prospective cohort study, which comprehensively assesses biomedical, psychosocial and behavioral risk factors [30]. Follow-up of a nationally representative sample of adult KT recipients from all six Swiss transplant centers occurs from pre-KT up to lifelong post-KT (6 months and 1 year post-KT, and yearly thereafter). Detailed information on the design of the STCS has previously been published elsewhere [30, 31]. The current study included data from patients enrolled between May 2008 (start of the cohort) and the end of 2017, who were aged ≥18 years at time of KT and had received a single-organ transplant. Follow-up of this cohort lasted until June 2019.

### Data Collection, Management and Ethics

The STCS was approved by the ethical committees of all Swiss KT centers [EKBB 351/07, KEK 270/07, EKSG 07/122, EK 1487, CER 07-301 (NAC 07-117), Lausanne 284/07]. After providing written informed consent, patients completed the pre-KT STCS Psychosocial Questionnaire to collect selected socio-demographic, psychosocial and behavioral data [31]. Data on recipients’ transplant outcomes (mortality and graft loss), age, and biomedical characteristics were collected from patient’s charts by local data managers.

### Variables and Measurement

Pre-KT covariates for the multivariable regression models, were based on evidence from the existing literature. We first determined the three controlling self-reported variables: depressive symptomatology, smoking and medication adherence of the STCS’s psychosocial framework that were collected since the beginning of the STCS [32–39]. The multivariable regression models included covariates—donor age, donor type, specific types of comorbidities (diabetes mellitus, cardiopulmonary comorbidity, cancer history) preemptive KT and total number of HLA mismatches—that have been routinely assessed by the STCS [3, 5, 6, 8, 13, 16, 20, 24, 28].

### Outcome Variables

Deaths recorded in the STCS were registered at the bedside by two physicians independently, and thereafter ascertained by the STCS endpoint committee. Graft loss as a primary cause of death is an unlikely event in the KT setting. To ensure correct classification of outcome events in patients with this primary cause of death registered in the STCS, their medical files were retrospectively re-analyzed by a physician of the transplant center where the patient was treated. Thereby, for patients who died due to multi-organ failure or a systemic infection (which secondary induced graft loss) a “*mortality*” *event* was considered as the first event. Mortality was recorded irrespective of previous graft loss, however, since graft survival cannot occur in patients already deceased, mortality was considered a competing risk of graft loss.

A *graft loss event* was defined as the absence of kidney function occurring at any time during follow-up, due to irreversible graft injury and requiring return to dialysis and/or re-KT. Death with a functioning graft was hereby not considered as graft loss.

*Self-rated health status* of the KT recipients was routinely assessed by the STCS at the time of listing, six, 12 months post-KT and then on a yearly base using the EQ VAS instrument, a PROM. The EQ VAS instrument is part of the EuroQol 5D instrument (EQ-5D), which is a preference-based measure of health status [40]. At each time point the EQ VAS score was collected by asking the KT-recipients to rate their self-perceived health today on a scale numbered from 0 to 100, where 0 represents the worst and 100 the best health they could imagine (continuous variable, presented as percentage). The EQ VAS instrument provides a quantitative measure of the patient’s perception of their overall health and therefore represents the patient perspective.

### Socio-Demographic, Behavioral and Psychosocial Characteristics

Socio-demographic characteristics extracted from the STCS baseline database included sex, race, marital status and age in years at transplantation. Depressive symptomatology pre KT was assessed with the 7-item depression subscale of the Hospital Anxiety and Depression (HADS) scale. Each HADS depression-subscale item was answered on a 4-point Likert scale (0 = “not at all” to 3 = “most of the time”), the total score was calculated by summing the item scores and used as a continuous variable (range 0–21) [31]. To assess implementation of medication adherence pre-KT two self-report items (taking adherence and drug holidays) from the Basel Assessment of Adherence to Immunosuppressive Medications Scale (BAASIS^®^) instrument—in an adapted version for adherence to other medications pre-KT—were used [31]. Medication non-adherence (yes/no) was defined as any missed doses, having missed at least one dose of medication and/or having missed two or more consecutive doses over the past 4 weeks. Psychometric data of the BAASIS^®^ were previously reported [41–44]. We assessed smoking through one self-report item on smoking status (yes/no).

### Biomedical Recipient, KT and Donor Characteristics

The STCS biomedical variables reflecting the recipient’s clinical status immediately pre-KT were: etiology of renal disease, type of renal replacement therapy received, years on dialysis, Anti-CMV status and pre KT comorbidities (cancer, diabetes mellitus or cardiopulmonary disease). Transplant-related variables were: type of KT (living, deceased-donor) date of KT (day/month/year), the total number of HLA mismatches, donors’ age in years and sex (female/male), delayed graft function (yes, no), reason for graft loss, described immunosuppressant (Table 1). Extended criteria donation was not reliably captured as a controlling variable for the models, as data collection was not conclusive.

**TABLE 1:** Sample characteristics.

Variable	Specification variable	Total sample (n = 2366)
**Outcomes**		
Mortality events	n (%)	298 (12.6)
Mortality events of patients without graft loss	n (%)	234 (9.9)
Graft loss events	n (%)	198 (8.4)
Time to death in months (n = 298)	Mean (SD)	45.9 (33.0)
	Median (IQR)	42.9 (54.0)
	Min—max	0.1–120.4
Time to graft loss in months (n = 198)	Mean (SD)	34.5 (31.6)
	Median (IQR)	28.6 (52.8)
	Min—max	0.0–118.0
Length of follow-up in months	Mean (SD)	72.0 (34.1)
	Median (IQR)	70.1 (61.2)
	Min—max	0.1–120.4
**Socio-demographic recipient characteristics**		
Age at transplantation	Mean (SD)	52.9 (13.6)
	Median (IQR)	55.0 (19.0)
	Min—max	18.0–82.0
Sex	Female, n (%)	848 (35.8)
Race	Caucasian, n (%)	2153 (91.7)
Marital status	Single, n (%)	378 (17.9)
	Married/living together, n (%)	1406 (66.7)
	Divorced/separated, n (%)	246 (11.7)
	Widow(er), n (%)	79 (3.7)
**Psychological and behavioral recipient characteristics**		
Depressive symptomatology^1^	Mean (SD) HADS score	4.5 (3.7)
	Median (IQR) HADS score	4 (4)
	Min—max	0–21
Medication non-adherence^2^	Yes, n (%)	677 (28.6)
Current smoking	Yes, n (%)	418 (19.6)
**Biomedical recipient characteristics KT and donor characteristics**		
Etiology of renal disease	Cause unknown, n (%)	136 (5.8)
	Congenital, n (%)	57 (2.4)
	Diabetic nephropathy, n (%)	195 (8.3)
	Glomerulonephritis, n (%)	561 (23.9)
	HIV nephropathy, n(%)	3 (0.1)
	Hereditary non PCKD, n (%)	76 (3.2)
	Interstitial nephropathy, n (%)	79 (3.4)
	Nephrosclerosis, n (%)	265 (11.3)
	Other, n (%)	283 (12.1)
	PCKD, n (%)	454 (19.3)
	Previous GF, n (%)	118 (5.0)
	Reflux/Pyelonephritis	120 (5.1)
Type of renal replacement therapy	None, n (%)	411 (17.4)
	Peritoneal dialysis, n (%)	319 (13.5)
	Haemodialysis, n (%)	1631 (69.1)
Years on dialysis	Mean (SD)	4.0 (5.0)
	Median (IQR)	3.0 (41.0)
	Min—max	1.0–42.0
Anti-CMV status	Seropositive, n (%)	1459 (61.9)
Cancer history	Yes, n (%)	258 (10.9)
Diabetes mellitus^3^	Yes, n (%)	651 (27.5)
Cardiopulmonary comorbidity^4^	Yes, n (%)	1180 (49.9)
**KT and donor characteristics**		
Type of KT	Deceased-donor, n (%)	1392 (58.8)
	Living-donor, n (%)	974 (41.2)
Extended criteria donation^5^ (n = 864)	Yes, n (%)	311 (36.0)
Total number of HLA mismatches^6^	Mean (SD)	3.8 (1.5)
	Median (IQR)	4 (2)
	Min—max	0–6
Donor age^7^	Mean (SD)	52.4 (16.1)
	Median (IQR)	55.0 (18)
	Min—max	0–88

1 Each HADS depression-subscale item was answered on a 4-point Likert scale (0=“not at all” to 3=“most of the time”), the total score was calculated by summing the item scores and used as a continuous variable (range 0–21).

2 Medication non-adherence (yes/no) was defined as any missed doses, having missed at least one dose of medication and/or having missed two or more consecutive doses over the past 4 weeks.

3 Defined as having diabetes mellitus 1 or 2 according to STCS definitions.

4 Defined as having coronary heart disease, cerebral vascular disease, peripheral vascular disease, left ventricular dysfunction according to STCS definitions.

5 Defined as a KT from a donor aged ≥60 years or aged ≥50 years with at least two of the following conditions: history of hypertension, serum creatinine >1.5 mg/dl or cerebrovascular accident as cause of death; 6 min 0; max 6.

6 Count of HLA mismatches.

7 Continuous variable in years since birth.

SD, standard deviation; IQR, interquartile range; HADS, Hospital Anxiety and Depression Scale; KT, kidney transplantation; STCS, Swiss Transplant Cohort Study; HIV, human immunodeficiency virus; PCKD, polycystic kidney disease; GF, allograft failure; Anti-CMV, anti-cytomegalovirus; HLA, Human Leukocyte Antigen.

### Data Analysis

Descriptive statistics were applied to describe the sample characteristics, perceived health status over time and the incidence of graft loss as well as mortality. Time to event analysis was performed by Cox’ proportional hazards regression analysis for mortality and graft loss, and—in the case of time to graft loss, also by competing risk analysis using *Fine and Gray’s* regression model [45]. A competing risk is defined as “an event whose occurrence either precludes the occurrence of another event under examination, or fundamentally alters the probability of occurrence of this other event” [27, 46]. The competing risk model estimates the prognosis of graft loss in the presence of mortality as a competing risk. Analyses for mortality and graft loss were executed unadjusted and adjusted for aforementioned controlling variables and additionally included examination of possible non-linear relationships of age with both outcomes by testing higher-order terms and also by plotting martingale residuals [47]. Missing data generally did not exceed 10%. However, in the case of non-adherence to medication with missing values of 14% of the sample resulting from the fact that not all wait-listed patients stated to be taking prescribed medications, we applied multiple imputation *via* “Multivariate Imputation by Chained Equations” (MICE). In this case MICE was performed in order to calculate adjusted models on the same sample as the unadjusted ones. Five rounds of “fully conditionally specificated imputation” were executed on variables deemed appropriate by the algorithm.

To analyse the relationship between age and self-rated health status, we applied a random-intercept regression model, predicting recipient’s repeatedly measured health status over time. Analyses were performed using SAS version 9.4 (SAS Institute Inc., Cary, NC, United States); MICE was performed in R 3.6.2 (cran.r-project.org). Alpha was set at *p* = 0.05.

## Results

### Sample Characteristics

**FIGURE 1:**
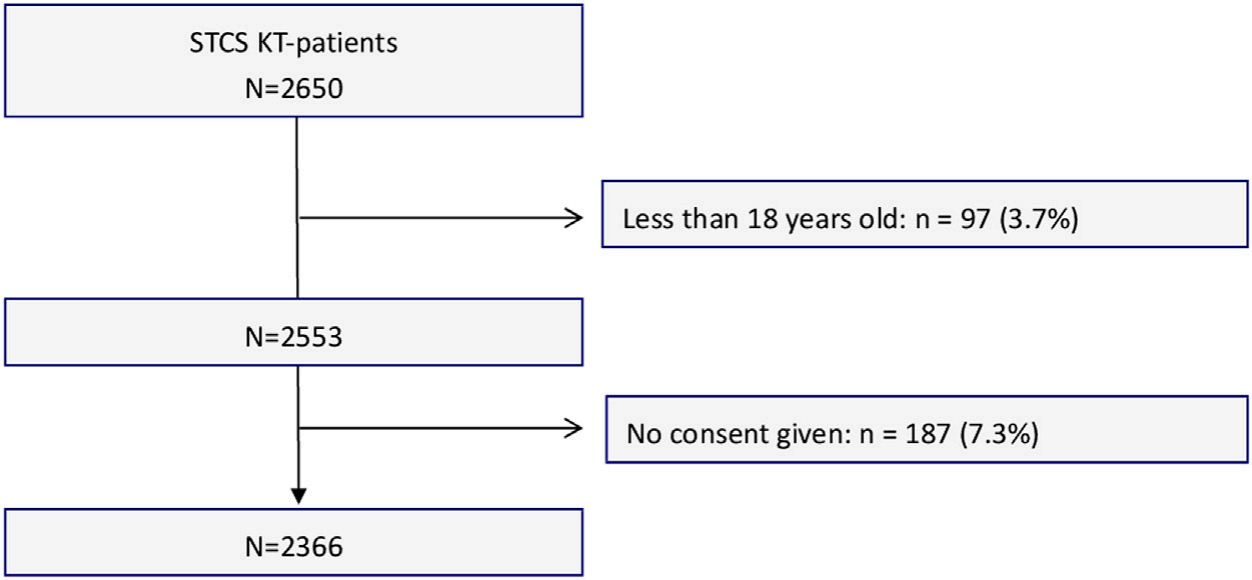
Flow chart of the sample composition.

For the current study, 2,553 KT recipients involved in the STCS were considered eligible, of whom 2,366 agreed and were included. A flowchart showing the sample selection process is provided in Figure 1. The sample’s median follow-up time (which lasted until June 2019) was 70.1 months (IQR 61.2, range: 0.1–120.4). We lost 27 patients to follow-up prior the end of the study period (n = 27, 1.1%).

Table 1 provides an overview of the sample characteristics. The average recipient age was 52.9 years (SD 13.6, range: 18–82) at the time of transplantation, 35.8% (n = 848) of the recipients were female. In 58.8% (n = 1392) of cases grafts were received from deceased donors, and the average donor age was 52.4 years (SD 16.1, range: 0–88). In our sample 8.4% experienced graft loss (n = 198) during the study period, 12.6% died (n = 298) and 2.7% experienced both outcomes (n = 64). The etiology of renal disease was glomerulonephritis (23.9%) and polycystic kidney disease (19.3%) in majority of studied patients. A non-adherence to the pre-KT medication was reported by 28.6% (n = 677) of the KT recipients and 19.6% (n = 418) were smoking at the time of transplantation. Renal replacement therapy before transplantation was provided for 69.1% (n = 1631) by haemodialysis treatment while 17.4% (n = 411) of KT recipients received a preemptive transplantation. We found a median HADS score of 4 (IQR 4, range: 0–21).

### Age at Time of Transplant and Mortality and Graft Loss

**FIGURE 2:**
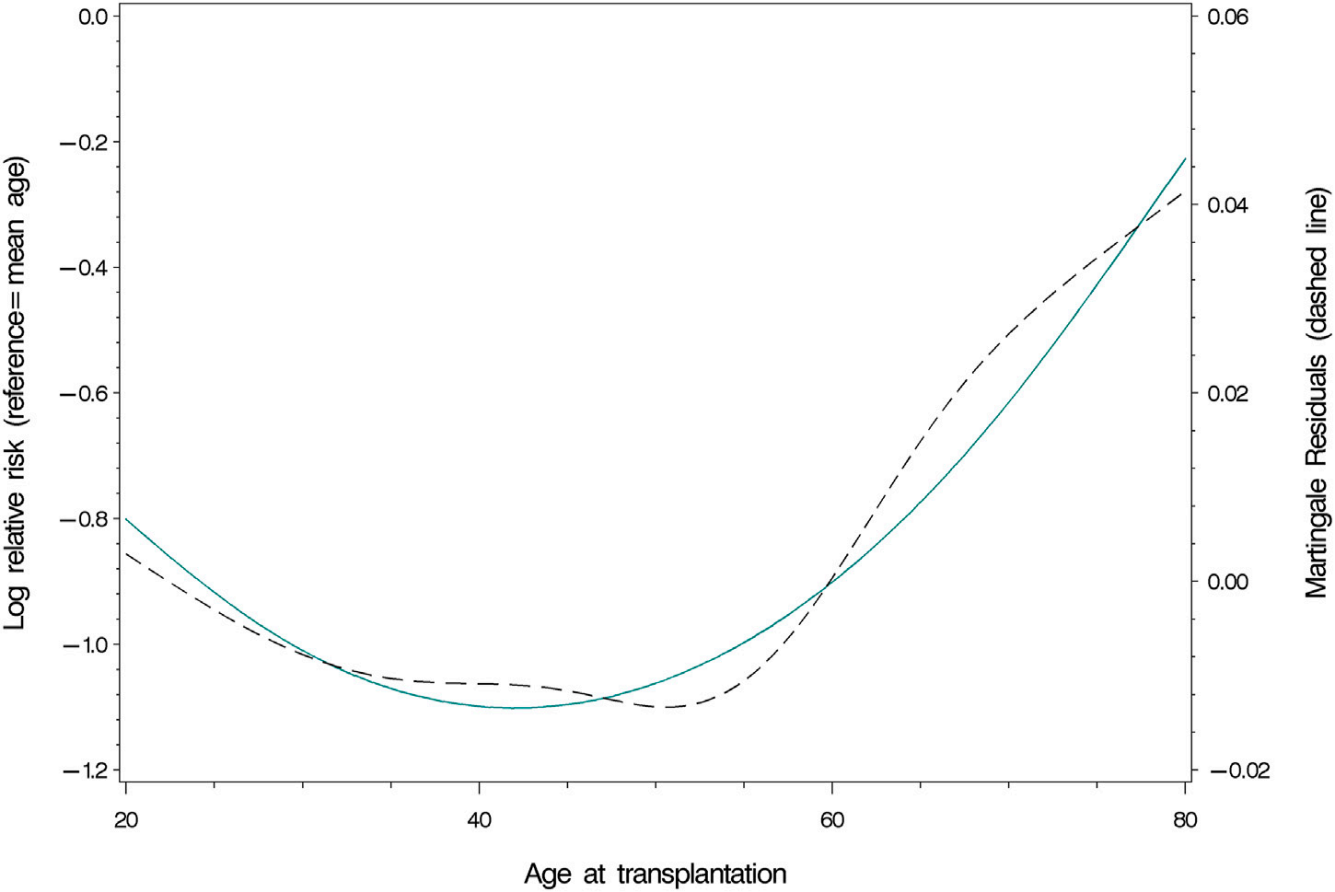
Nonlinear relationship between the probability of graft loss and age at transplantation.

Age at the time of transplantation predicted mortality in a linear fashion [HR (Hazard Ratio) = 1.07; 95% CI: 1.06–1.08; *p* <0 .0001; Table 2]. The relationship remained intact when adjusting for covariates (HR = 1.07; 95% CI: 1.05–1.08; *p* <0 .0001), of which current smoking (*p* = 0.0060), diabetes mellitus (*p* = 0.0056) and donor type (*p* = 0.0002, better survival for living donor grafts) were significant. Concurrently, age at time of transplantation predicted graft loss, though in a non-linear way (*p* = 0.0224; Table 2). Figure 2 displays the results of our examination of non-linear relationships and shows that patients between 35 and 55 years of age had a lower risk of experiencing graft loss, while the probability of those younger and older was higher. This non-linear relationship remained significant (*p* = 0.0224) after controlling for covariates. Recipients who received a transplant from a living donor (*p* = 0.0008) and preemptive KT (*p* = 0.0184) recipients experienced lower graft loss rates. Table 2 displays the results comparing statistical models using Cox’ regression and Fine and Grays’ competing risk approach, showing only negligible differences between the two analysis methods.

**TABLE 2:** Results of the survival analyses.

Outcome	Pre-KT predictor	Hazard ratio Cox’ regression (95% confidence interval)	*p*-value	Hazard ratio fine & gray model (95% confidence interval)	*p*-Value
Mortality	Unadjusted model	^1^			
	Age at KT	1.07 (1.06–1.08)	<0.0001	/	
	Adjusted model	^2^			
	Age at KT	1.07 (1.05–1.08)	<0.0001	/	
	Current smoking	1.48 (1.12–1.95)	0.0060	/	
	Medication adherence	0.88 (0.66–1.18)	0.4086	/	
	Cancer history	0.84 (0.60–1.17)	0.3012	/	
	Cardiopulmonary comorbidity	0.71 (0.26–1.94)	0.5024	/	
	Diabetes mellitus	1.40 (1.10–1.77)	0.0056	/	
	Depressive symptomatology	1.00 (0.97–1.03)	0.9541	/	
	Donor age	2.58 (0.92–7.17)	0.0704	/	
		Donor type^5^	0.69 (0.52–0.91)	0.0097	/	
		Preemtive KT	1.65 (1.04–2.64)	0.0344	/	
Graft loss	Unadjusted model	^3^			
	Age at KT	0.95 (0.89–1.01)	0.0949	0.95 (0.90–1.01)	0.1181
	Age^squared^ at KT	1.00 (1.00–1.00)	0.0457	1.00 (1.00–1.00)	0.0724
	Adjusted model	^4^			
	Age at KT	0.93 (0.88–0.99)	0.0241	0.94 (0.88–0.99)	0.0367
	Age^squared^ at KT	1.00 (1.00–1.00)	0.0224	1.00 (1.00–1.00)	0.0425
	Current smoking	1.33 (0.96–1.86)	0.0905	1.29 (0.93–1.81)	0.1335
	Medication adherence	0.88 (0.63–1.23)	0.4585	0.88 (0.63–1.23)	0.4696
	Cardiopulmonary comorbidity	1.15 (0.28–4.70)	0.8493	1.33 (0.34–5.18)	0.6843
	Diabetes mellitus	1.17 (0.86–1.60)	0.3145	1.14 (0.84–1.55)	0.4168
	Depressive symptomatology	1.00 (0.97–1.04)	0.8676	1.00 (0.97–1.04)	0.8673
	HLA mismatches	1.02 (0.92–1.13)	0.7752	1.02 (0.92–1.13)	0.7250
	Donor age	1.19 (0.28–4.90)	0.8131	1.00 (0.25–3.95)	0.9958
		Donor type^5^	0.55 (0.39–0.78)	0.0008	0.57 (0.40–0.80)	0.0013
		Preemptive KT	2.05 (1.13–3.71)	0.0184	2.03 (1.11–3.70)	0.0212

C-statistics (95%CI).

1 0.72 (0.69–0.74).

2 0.75 (0.72–0.77).

3 0.55 (0.51–0.58).

4 0.65 (0.61–0.69).

5 better survival for living donor grafts.

A sensitivity analyses was performed applying modeling without imputations, finding that the quadratic term that predicted graft loss was insignificant (*p* = 0.18), for which the adherence variable was responsible. However, this was not because of a confounding relationship of adherence, but resulting merely from the missing subjects, as omitting the same patients in the unadjusted model had the same effect. Results also show a collinearity between cardiopulmonary comorbidities and donor age; both were in all models statistically significant if included separately. Both variables were kept in the model, as they were not the primary aim of our analysis and were only needed as controlling factors.

### Age at Time of Transplant and Self-Rated Health Status

**FIGURE 3:**
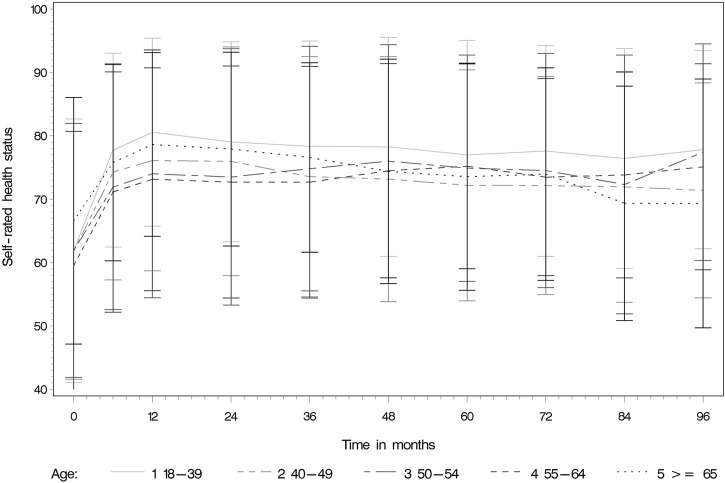
Self-rated overall health during the assessment period from pre-KT up to 8 years post-KT.

The median pre-KT health status was rated at 65/100 (IQR 30). The median self-rated health status was assessed noticeably higher during the whole post-KT follow up time (e.g., 12 months post-KT median EQ-VAS 80/100 IQR 21). Table 3 and Figure 3 display the self-rated health status during the assessment period from pre-KT up to 8 years post-KT, showing higher scores at all follow-up time-points compared to the pre-KT status, regardless of age. Generally, younger and older KT patients rated their health status higher before and after KT compared to middle-aged. No relationship of age with health status could be detected (Table 4) (*β* = −5.29; 95% CI: −3.36 to 0.85; *p* = 0.0844).

**TABLE 3:** Self-rated health status.

Month of follow up	N	Mean (SD) EQ-VAS	Median (IQR) EQ-VAS
Baseline	2098	62.2 (20.6)	65.0 (30.0)
6	1776	74.0 (17.5)	80.0 (22.0)
12	1633	76.2 (17.0)	80.0 (21.0)
24	1336	75.6 (17.6)	80.0 (20.0)
36	1107	74.9 (17.7)	80.0 (25.0)
48	863	75.1 (17.8)	80.0 (24.0)
60	669	74.5 (17.5)	80.0 (25.0)
72	509	74.1 (16.8)	79.0 (21.0)
84	349	72.6 (17.9)	76.0 (24.0)
96	190	74.0 (17.2)	80.0 (20.0)

EQ-VAS, EuroQol Visual Analogue Scale; SD, standard deviation; IQR, interquartile range.

**TABLE 4:** Results of the linear mixed-model regression analysis, predicting (square-transformed) health status.

Effect	Estimate (95%Confidence interval)	Standard error	DF	t Value	Pr > |t|
(1) Intercept	6445.31 (6107.74–6782.88)	172.15	2376	37.44	<0.0001
(2) Follow up time in months	−7.26 (−9.56 to −4.96)	1.1711	1203	−6.20	<0.0001
(3) Measurements from month 12 on (yes/no)	−1883.03 (−1984.47 to −1781.58)	51.7514	8755	−36.39	<0.0001
(4) Interaction effect of follow up time in months (2) with the binary variable before/from month 12 on (3)	250.42 (234.05 to 266.79)	8.3534	7829	29.98	<0.0001
(5) Age in years	−5.29 (−11.31 to 0.72)	3.0660	2191	−1.73	0.0844

Note: Parameters (3) and (4) were functional in modeling the initial increase in the health status curve as shown in Figure.

## Discussion

The objective of this prospective nationwide cohort study was to analyze the relationship between age at time of KT with mortality, graft loss incidence and self-rated health status in adult KT recipients. Age at the time of KT predicted mortality in a linear fashion but a non-linear relationship between age and graft loss was detected. Our analysis indicates that by taking into account the competing risk of mortality in estimating probabilities of graft loss, the risk of both outcomes is fairly independent of each other. Thus, graft loss probabilities can be reasonably well estimated using Cox’ regression analysis without applying a competing risk analysis. It should be noted that only 2.7% of our sample experienced both outcomes, and a higher overlap may result in larger differences between the two analysis methods. The self-rated health status during the assessment period from pre-KT up to 8 years post-KT showed higher scores at all follow-up time-points compared to the pre-KT status, regardless of their age. No relationship between age at time of KT and post-KT self-rated health status was found, age therefore did not predict this outcome.

Our results support those of previous studies that reported increased mortality after KT in older recipients compared to younger ones; however, this is consistent with data from the general population [12–16]. Whereas the linear relationship between age and patient survival in the current study does not back the assumptions of previous studies that stepwise mortality risk changes across age groups exist. The result of our analysis does hence not support the use of age as a categorical variable to interpret study findings of patient survival in KT. In contrast, the nonlinear prediction of graft loss by age at the time of transplantation does not reflect conclusions of studies that found a linear increase in post-KT graft loss with older patients [14, 17, 23]. Patients between 35 and 55 years of age presented with a lower risk of experiencing graft loss in our study, whereas older and younger recipients showed a higher probability. Pre-transplant drug non-adherence is a proposed factor that can negatively influence adherence to immunosuppressive regimen in transplant candidates. Several studies reported that non-adherence to the immunosuppressive regimen has a negative effect on graft and patient survival in the population of KT recipients [36–38, 48–51]. Evidence shows that younger adults are at greater risk for drug non-adherence in KT [37]. Concurrently, mild cognitive impairment and the presence of additional comorbidities are common in ESRD and KT recipients. They are also found to be associated with older age in these populations [49, 50]. Furthermore, mild cognitive impairment is associated with decreased medication adherence as well as health literacy in KT recipients [51]. This evidence may support our findings that middle-aged adults after KT have a lower risk of experiencing graft loss than their younger and older counterparts.

Chronological ageing alone has been described as an inaccurate representation of patients’ functional ability and individuals of similar age can show diverse physical and cognitive conditions [28, 52]. Biological age in turn was found to be a strong independent predictor for adverse health outcomes such as mortality and graft loss in KT recipients [5, 8, 28, 52]. Physical frailty is currently proposed as an indicator for biological age [5, 8, 28]. The inclusion of frailty measurements to determine the biological age of a KT recipient could hence be a valuable addition to the single determination of age counted in years in the KT population to predict adverse health outcomes. Relevant associations and organizations increasingly acknowledge the importance of the inclusion of frailty assessments in clinical practice guidelines for evaluating and managing candidates for KT [5, 8, 28, 53].

Besides biological age, psychosocial factors can independently predict poor post-KT outcomes and are increasingly valued in transplant research [31, 54]. International transplant societies endorse a comprehensive bio-psychosocial evaluation prior to transplantation and include them in their clinical guidelines [14, 20, 28]. With a low median HADS score of 4 points, our study participants reported fewer depressive symptoms than described in other studies [33, 55]. However, our study showed that 28.6% of the KT recipients were non-adherent to their medication before transplantation and 19.6% were smoking. These figures reflect the results of previous research [32, 34, 56] but only current smoking status was determined as a significant covariate for the mortality outcome event in our sample. No other psychosocial covariate was identified as significant in our analysis. These results could be due to the fact that in our current study only a limited number of psychosocial factors could be considered as covariates, since routine data collection of a comprehensive set of variables has only been added more recently. The STCS Psychosocial Questionnaire is self-administered and not conducted as a face-to-face interview.

Regardless of age, the self-rated health status during the whole follow-up period was rated notably higher post-KT. This shows that the effect of the intervention from a patient perspective was influencing their health status positively in a sustainable way and therefore KT presents a longtime advantage compared to the pre-transplant status. This finding concurs with previous smaller studies over shorter time periods showing an increase in quality of life and self-rated health post-KT [9, 26] and can be used in clinical practice for counselling particularly of older potential KT recipients. To include the patient perspective on health outcomes by assessing PROMs such as self-rated health status in the pre-KT evaluation and decision making should therefore be considered.

The strengths of this extensive study are its longitudinal, prospective design as well as the application of competing risk analysis. The nationwide multi center design in a European setting including a comprehensive sample of KT recipients with an extensive follow-up time, provides insights regarding relationships between patient’s age and the post-KT outcomes of patient and graft survival. The application of competing risk analysis allows the prognosis of graft loss in the presence of mortality as a competing risk. Despite its’ strong and rigorous study design, a notable weakness of this study is that only 2.7% of our sample experienced both outcomes of mortality and graft loss. A higher overlap may result in larger differences between the competing risk and standard survival analysis. Thus, in samples with a higher overlap, the probabilities of graft loss may not be sufficiently well estimated if only Cox’ regression analysis is used without applying a competing risk analysis. A further weakness of our study is that we assessed only a limited number of pre-KT psychosocial factors.

## Conclusion

This study revealed that age at the time of KT predicted mortality in a linear fashion concurring with records from the general population in the same country. In contrary, a non-linear relationship between age and graft loss was detected showing that KT recipients aged between 35 and 55 years presented with the lowest risk of experiencing a graft loss event. Taking into account the competing risk of mortality in estimating probabilities of graft loss, the risk of both outcomes was fairly independent from each other. Thus, graft loss probabilities can be estimated using Cox’ regression analysis. Self-rated health status during the follow-up period was indicated notably higher post-KT, regardless of age. No relationship between age at time of KT and post-KT self-rated health over the entire follow-up time was found. Therefore, age alone seems to be an inaccurate measure to guide risk prediction in KT. This underlines the importance of exploring further aspects such as biological age as a valuable addition to existing KT-guidelines aiming to provide pre-tailored and effective guidance particularly as individuals of similar age can show substantially diverse conditions.

## Capsule Sentence Summary

The numbers of older patients considered eligible for and undergoing kidney transplantation are increasing. However, the effect of age at time of transplantation on health outcomes in kidney transplantation remains inconclusive. The objective of our study was to analyze the relationship between age at time of kidney transplantation with mortality, graft loss and self-rated health status in adult kidney transplant recipients. We used data from the prospective Swiss Transplant Cohort Study and included data of 2366 kidney transplant recipients who received a single-organ kidney transplant between 2008 and 2017. Age at transplantation linearly predicted mortality. It was also predictive for graft loss, though nonlinearly, showing that recipients aged between 35 and 55 years presented with the lowest risk of experiencing graft loss. Self-rated health status during the follow-up period was indicated notably higher post-transplantation, regardless of age. No relationship of age with self-rated health status was detected. Therefore, age alone seems to be an inaccurate measure to guide risk prediction and clinical decision making in kidney transplantation. This underlines the importance of exploring further aspects such as biological age including cognition, psychosocial factors, PROMs and physical functioning as a valuable addition to existing KT-guidelines aiming to provide pre-tailored and effective guidance particularly as individuals of similar age can show substantially diverse conditions.

## Data Availability

The datasets presented in this article are not readily available because dataset cannot be shared due to current STCS policy. Requests to access the datasets should be directed to www.stcs.ch.

## Abbreviations

Anti-CMV, anti-cytomegalovirus; BAASIS^®^, Basel Assessment of Adherence to Immunosuppressive Medications Scale; ESRD, end-stage renal disease; EQ-VAS, EuroQol Visual Analogue Scale; GF, allograft failure; HADS, Hospital Anxiety and Depression Scale; HIV, human immunodeficiency virus; HLA, human leukocyte antigen; HR, hazard ratio; IQR, inter quartile range; KT, kidney transplantation; MICE, Multivariate Imputation by Chained Equations; PCKD, polycystic kidney disease; PROM, patient reported outcome measure; SD, standard deviation; STCS, Swiss Transplant Cohort Study.
