# Supplementary material for: Age at Time of Kidney Transplantation as a Predictor for Mortality, Graft Loss and Self-Rated Health Status: Results From the Swiss Transplant Cohort Study
Source: Transpl Int. 2022 Jan 27;35:10076. doi: 10.3389/ti.2021.10076 (PMC8842256; doi:10.3389/ti.2021.10076)
Supplement: Supplementary file 1 [file DataSheet1.PDF]

Appendix I

Members of the Psychosocial Interest Group of the STCS

Sabina De Geest, Kris Denhaerynck, Lynn Leppla, Janette Ribaut (University of Basel); Annette Boehler, Michael Koller, Lut Berben (University Hospital Basel); Nadine Beerli, Oliver Mauthner (University Department of Geriatric Medicine Felix Platter, University of Basel,), Uyen Huynh-Do (University Hospital Inselspital Bern);; Karine Hadaya (University Hospital Geneva); Annina Seiler (University Hospital Zurich); Isabelle Binet (Cantonal Hospital St. Gallen); Patrizia Künzler-Heule (University of Basel, Cantonal Hospital St. Gallen); Hanna Burkhalter (Kantonsspital Graubünden), Marian Struker (Kinderspital Zürich), Sonja Beckmann (University of Basel, University Hospital Zurich), Christian Rothlisberger (patient representative).

Active members of the Swiss Transplant Cohort Study (STCS)

Patrizia Amico, John-David Aubert, Vanessa Banz, Guido Beldi, Christian Benden, Christoph Berger, Isabelle Binet, Pierre-Yves Bochud, Sanda Branca, Heiner Bucher, Thierry Carell, Emmanuelle Catana, Yves Chalandon, Sabina de Geest, Olivier de Rougemont, Michael Dickenmann, Michel Duchosal, Laure Elkrief, Thomas Fehr, Sylvie Ferrari-Lacraz, Christian Garzoni, Paola Gasche Soccal, Christophe Gaudet, Emiliano Giostra, Déla Golshayan, Karine Hadaya, Jörg Halter, Dimitri Hauri, Dominik Heim, Christoph Hess, Sven Hillinger, Hans H. Hirsch, Günther Hofbauer, Uyen Huynh-Do, Franz Immer, Richard Klaghofer, Michael Koller (Head, Data Center), Bettina Laesser, Guido Laube, Roger Lehmann, Christian Lovis, Pietro Majno; Oriol Manuel, Hans-Peter Marti, Pierre Yves Martin, Michele Martinelli, Pascal Meylan, (Head, Biological Samples Management Group), Nicolas J. Mueller (Chairman, Scientific Committee), Antonia Müller, Thomas Müller, Beat Müllhaupt, Manuel

Pascual (Executive Office), Jakob Passweg, Klara Posfay-Barbe, Juliane Rick, Eddy Roosnek, Anne Rosselet, Silvia Rothlin, Frank Ruschitzka, Urs Schanz, Stefan Schaub, Aurelia Schnyder, Christian Seiler, Jan Sprachta; Susanne Stampf, Jürg Steiger (Head, Executive Office), Guido Stirnimann, Christian Toso, Christian Van Delden (Executive Office), Jean-Pierre Venetz, Jean Villard, Madeleine Wick (STCS Coordinator), Markus Wilhelm, Patrick Yerly.
